# Capillary refill time in sepsis: A useful and easily accessible tool for evaluating perfusion in children

**DOI:** 10.3389/fped.2022.1035567

**Published:** 2022-11-17

**Authors:** Shirley Lamprea, Jaime Fernández-Sarmiento, Sofía Barrera, Alicia Mora, Juan Pablo Fernández-Sarta, Lorena Acevedo

**Affiliations:** Department of Critical Care Medicine and Pediatrics, Universidad de La Sabana, Fundación Cardioinfantil-Instituto de Cardiología, Bogotá, Colombia

**Keywords:** perfusion, septic shock, mortality, children, microcirculation

## Abstract

The international sepsis guidelines emphasize the importance of early identification along with the combined administration of fluids, antibiotics and vasopressors as essential steps in the treatment of septic shock in childhood. However, despite these recommendations, septic shock mortality continues to be very high, especially in countries with limited resources. Cardiovascular involvement is common and, in most cases, determines the outcomes. Early recognition of hemodynamic dysfunction, both in the macro and microcirculation, can help improve outcomes. Capillary refill time (CRT) is a useful, available and easily accessible tool at all levels of care. It is a clinical sign of capillary vasoconstriction due to an excessive sympathetic response which seeks to improve blood redistribution from the micro- to the macrocirculation. An important reason for functionally evaluating the microcirculation is that, in septic shock, the correction of macrocirculation variables is assumed to result in improved tissue perfusion. This has been termed “hemodynamic coherence.” However, this coherence often does not occur in advanced stages of the disease. Capillary refill time is useful in guiding fluid resuscitation and identifying more seriously affected sepsis patients. Several factors can affect its measurement, which should preferably be standardized and performed on the upper extremities. In this review, we seek to clarify a few common questions regarding CRT and guide its correct use in patients with sepsis.

## Introduction

Sepsis and septic shock continue to be the main causes of death in children. The global incidence of sepsis for the general population in 2017 was estimated to be 48.9 million cases, with 11 million deaths (much more than the population of Arizona, for example) (1). Almost half of these cases involved children under the age of five (20 million cases with 2.9 million deaths). In the United States, in adults, sepsis is a more frequent cause of hospitalizations than acute myocardial infarction and cerebrovascular disease and contributes half of inpatient deaths (2). These outcomes, their sequelae and complications may be more common in middle and low-income countries (2). Due to its disease burden, sepsis has been recognized as a global health priority by the World Health Organization (3).

Early recognition of hemodynamic abnormalities with bedside measurement in the different areas of the hospital is fundamental for improving outcomes. Capillary refill time is one of these tools for evaluating tissue perfusion which is easily available, easy to perform, and has no additional cost for patients with sepsis. Capillary refill time refers to the time it takes the nail bed of a distal phalange to return to its normal color after pressure has been applied with the extremity at heart level (4–6). It was first introduced by Beecher et al. in 1947 as a way of evaluating circulatory status and perfusion in critically injured soldiers, in order to evaluate and predict the severity of their injuries (7).

Today, the sepsis guidelines, American Academy of Pediatrics, World Health Organization (WHO) and American Heart Association recommend CRT measurement as part of a systematic assessment (2–5). It is part of the clinical assessment of hemodynamic involvement and tissue perfusion. An abnormal CRT has been associated with organ dysfunction, complications and greater mortality (6). However, there are several factors that can affect its measurement, a clear cut-off point has not been established, and its advantages over some biomarkers of tissue perfusion like lactate are often unknown. In this review, we use a series of questions to address the current uncertainties in daily clinical practice regarding the use of CRT in children with sepsis.

## Is the pathophysiological reason for a prolonged capillary refill time a tissue perfusion abnormality? *true*

The main function of the cardiovascular system is to maintain a supply of oxygen and nutrients to the tissues (7–11). However, in conditions like sepsis and different stages of shock, the relationship or coherence between the micro- and macrocirculation is lost as the disease advances (4, 9). In this regard, studies have found no relationship between heart rate or blood pressure and microcirculation in the advanced stages of sepsis, a phenomenon which has been termed “*dynamic incoherence*” (9). Any organ may be affected, including the kidneys, skin and nervous system. Their assessment is useful for taking therapeutic measures and may be done using clinical or metabolic parameters or minimally invasive measurements (Table 1).

**Table 1:** Description of perfusion evaluation methods.

Evaluation	Method	Cut-off point	Advantage	Disadvantage	Reference
Clinical evaluation	Body temperature		Low cost and easy implementation Allows assessment of “cold” or “warm” shock	Requires an assessment tool; is dependent on room temperature	(4, 5)
	Temperature difference	Skin temperatura difference assessed using the back of the hand	Low cost and easy implementation	Observer-dependent	(4, 5)
	Skin findings	Pallor, cyanosis, skin mottling	Low cost and easy implementation Microcirculatory evaluation Interobserver agreement	Difficult to evaluate in people with dark skin	(4, 5, 8)
	Capillary refill time	<2 s	Low cost and easy implementation Quantification of capillary perfusion Microcirculatory approach	Depends on room temperature, skin color, age	(5)
Metabolic evaluation	Lactate	<2 mmol	Low cost and easy implementation Flow-sensitive parameter	Increased lactate with beta-adrenergic stimulation	(4, 9)
	SvmO2—ScvO2	70%	Continuous and/or intermittent measurement Interchangeable for clinical purposes Flow-sensitive parameter	SvmO2 requires blood drawn from a pulmonary artery catheter, while ScVO2 can be easily obtained from a central venous catheter	(4, 9)
	Venous-arterial CO2 difference	6 mmHg	Flow-sensitive parameter	Intermittent measurements, need for arterial and venous samples	(4, 9)
Evaluation with imaging	Doppler		Flow assessment using Doppler	Requires equipment and trained staff	(4)
Other evaluation methods	NIRS		Non-invasive	Requires equipment Evaluate limitations: edema, jaundice	(4)
	Video-microscopy	Depends on the approach	Direct visualization of the microcirculation	Result variability Requires equipment	(4, 10)

Svm02: mixed venous oxygen saturation. Scv02 venous oxygen saturation. NIRS: Near-infrared spectroscopy.

**Figure 1:**
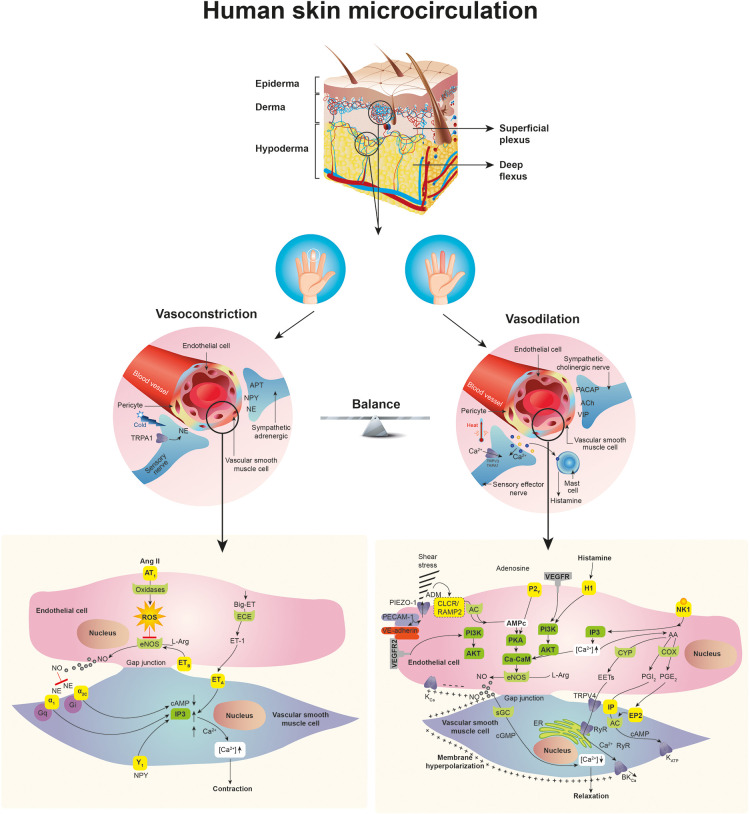
Skin characteristics of microcirculation and factors affecting vascular response.

The skin is the most accessible organ for evaluating perfusion without the need for instruments (4, 12). The superficial layer, or epidermis, is dense and avascular, and its main function is to protect and waterproof the underlying organs (12). The skin's nourishment and metabolic exchange occur in the dermis, which is made up of collagen and elastic fibrils embedded in a mucopolysaccharide matrix. The skin is an organ responsible for thermoregulation, among other functions, which is mainly controlled by the sympathetic nervous system (11, 12). Normal cutaneous microcirculation is organized in two plexuses parallel to the skin surface: the upper plexus and lower plexus. The upper plexus, located in the papillary dermis, plays a role in the skin's nourishment and consists of small arterioles and venules with an approximate external diameter of 20 μm and capillary loops that extend perpendicularly to the skin's surface and are 1 mm away from the surface (12). The lower plexus, in turn, is made up of arteries and veins which arise from the underlying muscle and adipose tissue. Human skin contains specific thermoregulatory structures made up of spiral vessels with thick, densely innervated walls, which connect the arterioles to venules in the dermis (12) (Figure 1).

From an anatomical standpoint, the skin and systemic microcirculation comprises the blood vessel zone from the arterioles to the venules and the capillary network between them made up of vessels measuring less than 20 microns (12). Both the arterioles and venules have vascular smooth muscle cells capable of responding to different mediators and regulating capillary blood flow (12). The capillaries are the most important site for delivering the necessary nutritional (gas exchange, water, nutrients and waste products) and non-nutritional (temperature control and immunological function) components to the tissues, and are considered to be those vessels under 10 microns in size containing a single file of red blood cells (12).

Functional capillary density and the number of capillaries in an area are determined by the volume occupied by the red blood cells (average size: 8 microns). At the same time, there are other associated metabolic factors in sepsis which alter capillary blood flow, such as arteriolar tone and capillary permeability (10). The first depends on a fine balance between vasocontrictive (noradrenaline, angiotensin II, vasopressin, endothelin 1 and thromboxane A2) and vasodilatory (prostacyclin, nitric oxide and adenosine) mediators (Figure 1) (6, 10). Under normal conditions, only 25% of the capillaries are active. A 50% decrease in the diameter of the capillaries smaller than 20 microns is thought to decrease blood flow by approximately 94% of its normal value. Capillary permeability refers to the leakage of various molecules and solutes due to the active disruption of the vascular barrier (which is different in each organ and depends on the type of associated noxa) and has proven to be better than other indicators like lactate in predicting the response to interventions (13, 14).

When a hemodynamic insult occurs, tissue perfusion changes are made to maintain an adequate blood flow in the macro- and microcirculation (9, 11). The associated sympathetic response is vasoconstriction, caused by noradrenaline release in the blood vessels, affecting the vascular smooth muscle cells. The *α*1 adrenergic receptors are stimulated, activating Gαq protein and, subsequently, the phospholipase-C-dependent signaling pathways, which allow calcium to enter the cell. All of this leads to vascular smooth muscle cell contraction (Figure 1) (11, 12). This phenomenon is more marked and evident in the skin due to the anatomical characteristics of the upper and lower plexus, as previously described. The consequence of this phenomenon is a decreased diameter and reduced blood flow in the smallest blood vessels of the microcirculation (8). The opposite phenomenon is also seen in patients with sepsis. What is known as “warm shock” is capillary bed and skin vasodilation related to poor noradrenaline release and a predominance of vasodilating substances like nitric oxide or prostaglandins. The ultimate goal is to maintain adequate blood flow and a balance between macro- and microcirculation.

## Can capillary refill time signal microcirculation disorders? *limited data*

An evaluation of perfusion and of microcirculation disorders is essential in the clinical approach to critically ill patients. Recent studies suggest that microcirculatory and endothelial abnormalities are the common basis of organ failure in sepsis (14). In septic conditions, the accumulation of proteases accelerates endothelial glycocalyx degradation, resulting in increased leucocyte adhesion to the vessel wall, increased vascular permeability and intravascular coagulation (15). This whole response leads to a profound disruption of tissue perfusion.

**Figure 2:**
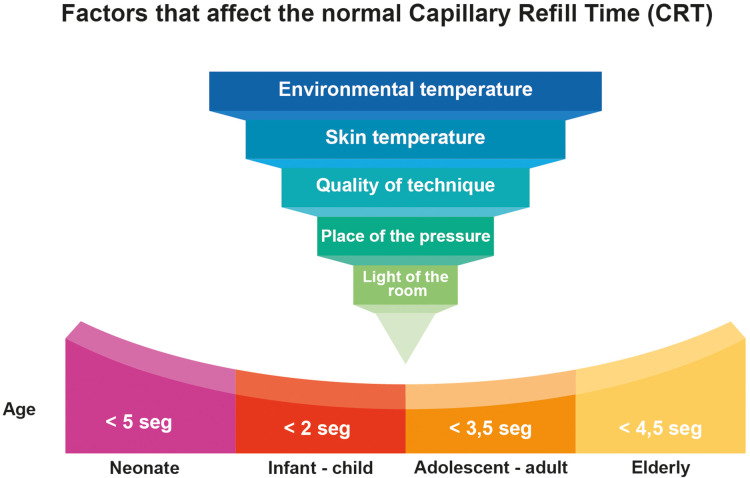
Factors that affect the Normal capillary refill time (CRT).

In the skin, poor peripheral perfusion represents a high adrenergic tone and low blood flow in the microcirculation. Recently, Valenzuela et al. evaluated the microvascular response to hyperoxia in healthy patients and those with sepsis (14). They simultaneously evaluated microcirculatory changes with sublingual video microscopy, the CRT and the difference between the core and peripheral temperature. In patients with sepsis, changes were found in microvascular flow heterogeneity after hyperoxia, with no changes in capillary density or CRT. In healthy subjects, no changes were observed in video microscopy findings nor in CRT. This differential response between patients with sepsis and healthy subjects can be magnified by the degree of adrenergic response and blood volume in systemic vasodilation states. In this regard, CRT is indicative of the adrenergic response and blood perfusion in sepsis, but may be affected by the severity and extent of the illness as well as other factors which will be explained further on (Figure 2) (16).

## Is there a standard method for measuring capillary refill time? *false*

**Figure 3:**
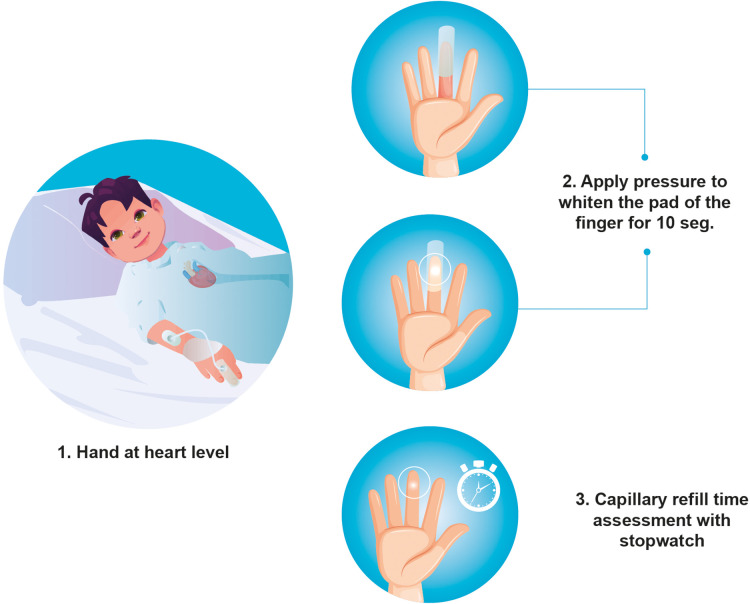
Suggested way to measure standardized capillary refill time in children.

Capillary refill time, as defined previously, can be measured in different ways. There are no studies comparing the various measuring techniques nor the ideal site for measuring it. Capillary refill time measurement requires visual inspection of blood returning to the distal capillaries after having been emptied by applying pressure (6). The site selected for measuring CRT (for example, the finger, hand, foot or chest) may lead to significantly different values (16). To assess CRT in healthy children, moderate pressure should be applied to the index finger for 5–10 s, using a timer to measure CRT and provide greater reliability (Figure 3) (16).

Hernández et al. standardized CRT measurement in their ANDROMEDA-SHOCK study (17). In this study, the measurement was taken by applying firm pressure with a glass microscope slide over the ventral surface of the distal phalange of the right index finger. Pressure was applied until the skin was white-pale and was held for 10 s. The time required to recover the baseline color after removing the pressure was recorded with a stopwatch as the CRT value. In this study, a capillary refill time greater than three seconds was considered abnormal (17).

Recently, Sheridan et al. (18) evaluated an automated device (Promedix Inc^©^) which uses a sensor on the patient's distal phalange to evaluate perfusion. After applying pressure and using algorithms, the exponential drop in the photodiode signal from the light source is calculated. The change in capillary flow timed with the pressure sensor can calculate the duration of capillary filling. After applying it in various critical care settings with septic patients, it was found to correlate well (*r* = 0.69) with the traditional method. The advantage of this system is that it would allow CRT measurement to be standardized through the use of technology and decrease variability and measurement errors (18, 19).

## Is the Normal range for capillary refill time less than two seconds for all ages? *false*

Various groups and organizations, including the World Health Organization, American Academy of Pediatrics, and American Heart Association consider CRT to be an easy, simple and non-invasive tool for evaluating the circulation, and one of the clinical elements for evaluating patients in shock. However, there is no consensus on the cut-off point for normal values. Historically, ever since the description by Beecher et al. in 1947, a normal value has been considered to be less than two seconds (7, 20, 21). However, this value has not been universally accepted, and healthcare staff's interpretations have been highly variable. Lobos AT et al. found that only 18% of staff physicians reported measuring CRT and 100% of those interviewed considered less than three seconds to be normal (20).

Establishing the same cut-off point for all ages and clinical conditions may lead to errors (Figure 2). In a systematic review of 21 studies including 1,915 children, Fleming et al. found that when CRT is standardized using moderate pressure on the index finger for five seconds and measured at a room temperature of 20°C–25°C, a value greater than three seconds should be considered abnormal (11). However, age is not clarified nor taken into consideration. As a predictor of mortality, in this study, CRT had a sensitivity of 34.6% (95% CI 23.9%–47.1%) and a specificity of 92.3% (95% CI 88.6%–94.8%). Overall, in children, a CRT of less than two seconds would rule out a serious condition, 2–3 s would require monitoring and a more comprehensive evaluation, and more than three seconds would be associated with greater mortality and worse outcomes in patients of all ages with sepsis (11).

In newborns (up to seven days old), the upper limit of normal may be 5–7 s (16), which is related to their skin immaturity. With these limits, a sensitivity of 55% and a specificity of 81% have been reported for detecting low blood flow (22–24). In healthy children, a normal CRT of approximately two seconds or less has been recorded when measured on the index finger, or four seconds or less if measured on the foot or chest (16). Studies in adults have found a wider variation, with an average time of 1.9 s (95th percentile 3.5 s) (21), 7% lower in men than women, and an average 3.3% increase in seconds per decade of life (6, 21) (Table 2).

**Table 2:** Normal CRT ranges.

Age group	Normal capillary refill time	Comments	Reference
Newborns (<7 days)	Up to 5–7 s	Newborn skin immaturity 55% sensitivity and 81% specificity for detecting low blood flow	(6, 22)
Infants and children	<2 s (measured in the index finger)	Five seconds of pressure on the index finger and at a temperature between 20°–25°C	(5, 11, 26)
	<4 s (foot or chest)	Same conditions as above	(16)
Adolescents and adults	<3.5 s		(21)
Older adults	<4.5 s	Related to vascular changes	(21)

## Are there factors that can interfere with the interpretation of capillary refill time? *true*

Capillary refill time is susceptible to factors that can profoundly affect the results, such as room temperature, the temperature of the skin, the patient's age, the room lighting, the quality of the technique and the pressure site (Figure 3). Some of these factors can be controlled, thus reducing technique variability (25).

Regarding the influence of temperature on CRT, it has been reported that, in healthy children in a warm environment, the normal CRT should be less than two seconds. The amount of light also affects the measurements. Under good conditions, 94.2% of the participants were reported as normal, compared with only 31.7% of the same participants in dim lighting (22).

Anderson et al. evaluated the environmental factors affecting CRT in adults. They found that the temperature of both the patient and the room modified CRT. For every 1°C decrease in the patient's temperature, the CRT increased 5%, regardless of the room temperature (21). The use of high-dose vasopressors has also been reported to affect the results (7).

Interobserver variability in measuring CRT should be taken into account, which has been reported to be up to 1.94 s, depending on the measuring environment (4). Brabrand M et al. found that the CRT interobserver variability among a group of nurses participating in the study was 0.62 (95% CI: 0.32–0.92) (26). This group of professionals is the one that most often (90%) reports the CRT in their charting, and doctors are the ones who least report it (18%) (20).

## Can capillary refill time be used as a resuscitation goal? *true*

Peripheral perfusion is an expression of the microcirculatory dysfunction in advanced stages of septic shock. It tends to be correlated with elevated lactate and multiple organ failure. In fact, microcirculation and endothelial abnormalities are considered to be the common denominator in multiple organ dysfunction syndrome in sepsis, as mentioned previously (8, 12, 15).

Bakker et al. evaluated peripheral perfusion in patients with sepsis, using clinical and metabolic parameters. They found that normal CRT values at six hours were independently associated with successful resuscitation (*p* = 0.02), and that this was the first hemodynamic parameter to return to normal (8). However, normalized central venous saturation (ScvO2) and the venous-arterial CO2 difference at six hours (considered separately and jointly), were not associated with a lactate value under 2.0 mmol/L. This study concluded that CRT recovery could predict successful resuscitation in the first 24 h after the intervention, in patients with circulatory dysfunction related to sepsis (8).

The ANDROMEDA-SHOCK study was a multicenter randomized clinical trial comparing fluid resuscitation guided by standardized capillary refill vs. serum lactate in patients with septic shock. The hypothesis of this study was that CRT-guided resuscitation would be associated with better results. The first analysis showed no difference in terms of mortality between the two groups (34.9% in the CRT group vs. 43.4% in the lactate group; HR 0.75 95% CI, 0.55–1.02; *p* = 0.06) (17). However, a *post hoc* analysis showed that patients whose treatment was guided by CRT required less fluid (*p* = 0.004) and fewer vasopressors (*p* = 0.001) and had a lower risk of death than those whose treatment was guided by lactate (OR 3.3; 95% CI 1.5–7.1; *p* = 0.003) (13). It is important to note that this post-hoc analysis controlled other factors that may have affected the initial results, and we believe CRT can be very useful for guiding interventions in these patients with septic shock.

In this regard, Lara et al. found that patients with an abnormal CRT had a greater risk of adverse outcomes [88% vs. 20%; RR 4.4 (2.7–7.4); *p* < 0.01] and inpatient mortality [63% vs. 9%; RR 6.7 (2.9–16); *p* < 0.001] than those with a normal CRT after fluid resuscitation with crystalloids. In fact, patients with a normal CRT after fluid administration had a greater probability of normalizing or decreasing their lactate levels than those with an abnormal CRT (77% vs. 38%, *p* = 0.01) (25).

## Is prolonged capillary refill time associated with abnormal organ perfusion in patients with sepsis? *true*

Capillary refill time, like other clinical parameters such as mottled skin, is an indicator of regional blood flow and has been described as an ultrasound substitute for vascular tone in visceral organs during early shock (8, 27). In the stages of shock, peripheral and visceral organ perfusion is reduced due to sympathetic nervous system activation, seeking a redistribution of blood flow to vital organs like the brain, heart and lungs (11, 12). Brunauer et al. studied the correlation in 30 adult septic shock patients between peripheral perfusion and the vascular tone of organs like the liver, spleen and kidneys, measured with the pulsatility index using Doppler ultrasound. They found an association between this index and CRT in abdominal organs. No association was found between the peripheral temperature and these indices measured by ultrasound. They concluded that CRT in adult patients may be correlated with ultrasound findings and may be a good surrogate for visceral organ vascular tone in the early stages of septic shock (27).

## Is capillary refill time associated with greater mortality? *true*

Possibly one of the greatest benefits of CRT is that it may be very useful for predicting patients with unsatisfactory outcomes, especially the groups with the greatest risk of death. Both in adults as well as in children, a CRT greater than three seconds is associated with worse outcomes (23, 28). Some authors have called it the “red flag” of hemodynamic assessment in sepsis. An abnormal finding under adequate measurement conditions with an adequate environmental temperature should alert the teams to an unstable patient with a potential for greater deterioration and worse outcomes.

In this regard, Bakker et al. found that an abnormal CRT after the initial fluid resuscitation was associated with an almost six times greater mortality compared with patients with a normal CRT after the initial fluid resuscitation (8). Fleming et al. described similar findings, with critically ill children with a prolonged CRT having a four times greater risk of death than patients with a normal CRT (23). In fact, an abnormal CRT could help define a phenotype with a greater or lower response to crystalloids and guide fluid resuscitation more quickly and effectively than lactate. These measures would have a positive impact on mortality and outcomes in patients with sepsis (29).

Capillary refill time is a reliable measure of mortality, regardless of the patient's disease stage and vasopressor support. Ait-Oufella et al. found that, in septic patients who received norepinephrine [0.3 mcg/kg/min (IQR 0.1–0.6)] and epinephrine [0.2 mcg/kg/min (IQR 0.02–0.2)] support, CRT was a good predictor of 14-day mortality, regardless of the type and intensity of vasopressor support they received. They also found that it was correlated with lactate and the SOFA score (30). The classification of “warm” or “cold” shock has recently been questioned by the sepsis guidelines (12, 30). A study of 469 patients with sepsis by Walker S et al. found that 65% were classified by the doctors as warm shock and 35% as cold shock. However, there was no concordance between the different clinical signs used to evaluate this classification (extremity temperature, CRT, pulse strength, pulse pressure and diastolic blood pressure) nor with the need for vasopressor support. Capillary refill time was associated with the type of shock after controlling for confusion (aOR 15.7; 95% CI 7.9–31.3). Shock was classified as “cold” or “warm” prior to 1960, when the pulmonary artery catheter was introduced in humans. This invasive monitoring coupled with radionuclide cineangiography showed that cardiac output could be maintained or even increased despite myocardial dysfunction and a reduced ejection fraction (31). These findings have suggested that all clinical signs of septic shock, including cardiovascular signs, are very complex and do not allow a classification based solely on the physical exam.

## Conclusions

In patients with septic shock, tissue hypoperfusion can be evaluated at the bedside using simple, easily accessible and universally applied clinical indicators such as CRT. For initial fluid resuscitation as well as follow up, this clinical tool provides a rapid and real-time guide to the hemodynamic support needs of patients in septic shock. Due to its characteristics, CRT may be affected by many environmental, age and support intensity factors, and therefore should be measured in a standardized manner and preferably in the upper extremities. The use of new technologies like digital videography or oxygen saturation probes will help decrease this test's rate of false negatives and will allow for an automated and standardized CRT measurement.
